# Transcriptomics of severe COVID-19

**DOI:** 10.18699/vjgb-26-08

**Published:** 2026-03

**Authors:** A.A. Gusarova, E.A. Trifonova, A.A. Babovskaya, M.M. Gavrilenko, V.A. Stepanov

**Affiliations:** Research Institute of Medical Genetics, Tomsk National Research Medical Center of the Russian Academy of Sciences, Tomsk, Russia; Research Institute of Medical Genetics, Tomsk National Research Medical Center of the Russian Academy of Sciences, Tomsk, Russia; Research Institute of Medical Genetics, Tomsk National Research Medical Center of the Russian Academy of Sciences, Tomsk, Russia; Research Institute of Medical Genetics, Tomsk National Research Medical Center of the Russian Academy of Sciences, Tomsk, Russia; Research Institute of Medical Genetics, Tomsk National Research Medical Center of the Russian Academy of Sciences, Tomsk, Russia

**Keywords:** severe COVID-19, RNA sequencing, transcriptomics, bulk RNA-seq, single-cell RNA-seq, differentially expressed genes, тяжелая форма COVID-19, секвенирование РНК, транскриптомика, bulk RNA-seq, single-cell RNA- seq, дифференциально экспрессирующиеся гены

## Abstract

Transcriptomics of severe COVID-19

## Abstract (ru)

Транскриптомика тяжелой формы COVID-19

## Introduction

COVID-19 (Coronavirus Disease 2019) is an acute respiratory infection caused by SARS-CoV-2. The clinical picture of COVID-19 has a wide range of manifestations and the course of the disease: it varies from mild to severe and critical (Prevention… COVID-19, 2023). The severe form may be accompanied by the development of acute respiratory distress syndrome (ARDS), which is characterized by an acute onset, severe hypoxemia, bilateral infiltration and pulmonary edema. Most patients with the severe form have lymphopenia, and some have thromboembolic complications. Severe COVID-19 can lead to multiple organ failure and death (Berlin et al., 2020; Huang C. et al., 2020).

Elucidating the pathogenetic basis of the disease, as well as key immune and inflammatory processes that differentiate a severe case from non-severe cases, is required to determine therapeutic strategies and prevention of severe COVID-19 (Jovic et al., 2022). Currently, laboratory blood tests are used for early identification of patients at risk of severe COVID-19, which can also be sensitive predictors of an unfavorable outcome of the disease (Chen et al., 2022; Roessler et al., 2023). In addition, the identification of such markers can contribute to the timely diagnosis of complications, monitoring and determination of optimal treatment (Tabassum et al., 2021). Biomarkers used to predict the severity of COVID-19 include complete blood count (white blood cell levels, levels of lymphocytes, neutrophils, neutrophil/lymphocyte ratio, platelet levels), markers of inflammation and proinflammatory cytokines (C-reactive protein, procalcitonin, ferritin, IL-6, IL-8, TNF-alpha, etc.), markers of damage to target organs (blood clotting factors, D-dimer, cardiomarkers and markers of kidney function), as well as markers of oxidative stress (levels of reactive oxygen species and antioxidants: vitamin C, thiol proteins, serum parameters of the glutathione system, redox status, lipid peroxidation) (Polonikov, 2020; Pincemail et al., 2021; Tabassum et al., 2021; Chen et al., 2022; Karu et al., 2022; Roessler et al., 2023; Liu X. et al., 2024). These biomarkers are significant indicators of severe COVID-19, but they are often identified only during the advanced stages of the disease (Chen et al., 2022). In this regard, studies aimed at identifying potential biomarkers that can be measured at earlier stages of infection and can predict the features of the organism’s immune defense against SARS-CoV-2 are becoming relevant (Schultze, Aschenbrenner, 2021).

Blood transcriptomics plays a significant role in determining the mechanisms that reflect the immune responses of the host organism. The study of transcriptomic profiles of patients with varying severity of infection can provide unique information about the biological processes underlying the severity of the course, which can be used to identify prognostic and diagnostic RNA markers, as well as therapeutic targets (Lee H.J. et al., 2018; Huang W. et al., 2021; Schultze, Aschenbrenner, 2021). Today, the most common method of transcriptome analysis is high-throughput RNA sequencing (RNA-seq), which is a quantitative system for genome-wide expression profiling and, therefore, is applicable to characterize events related to dysregulation of gene expression in patients with severe COVID-19 (Hegenbarth et al., 2022).

Differences in gene expression identified in different clinical severity of the disease indicate that there are potential genes associated with the progression of the disease, rather than with the disease as a whole. Identification and functional characterization of differentially expressed genes in severe and moderate forms of the disease seem to be a promising strategy for identifying biomarkers of the severity of COVID-19. This can provide a deeper understanding of the pathogenesis of COVID-19, helping in the choice of treatment methods by deciphering the complexity of the immune response of organism and discovering new therapeutic targets (Arunachalam et al., 2020; Bando et al., 2023). However, reliable RNA markers have not been established in clinical practice to identify patients who may develop severe COVID-19.

Therefore, in this review, we focused on the search for shared genes when analyzing the differential expression of genes obtained in the study of moderate and severe forms of COVID-19 using leukocyte RNA sequencing.

## Analysis of whole blood transcriptome in the various COVID-19 severity

**Fig. 1.**
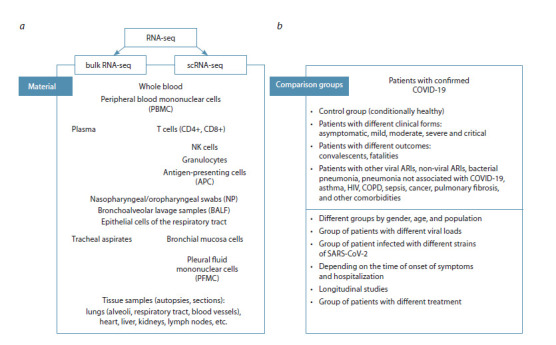
RNA sequencing approaches, materials (a) and comparison groups (b) in the study of moderate and severe forms of COVID-19.

Up to 2025, multiple transcriptome COVID-19 studies have been conducted. The number of different datasets in the GEO database (https://www.ncbi.nlm.nih.gov/geo/) is about 300, whereas there are more than 60 original studies in the PubMed database. The design of such studies includes various RNA sequencing approaches, comparison groups, and analyzed materials (Fig. 1).

The main sample type for gene expression profiling is peripheral blood and its components. In connection with the main clinical manifestations of COVID-19, transcriptomes of nasopharyngeal/oropharyngeal swabs (Chua et al., 2020; Hadzega et al., 2024), bronchoalveolar lavage fluid (BALF) samples (Xiong et al., 2020; Nassir et al., 2021), etc. are also being studied. Since the entry receptor for SARS-CoV-2 is found in many tissues (https://www.uniprot.org/uniprotkb/ Q9BYF1/entry), the effect of coronavirus infection on other organ systems is being investigated, including the analysis of transcriptomes of heart, liver, kidney, and lymph node tissues (Delorey et al., 2021), as well as the main target of SARSCoV- 2: lung tissue (Fig. 1a). The main comparison groups include patients with confirmed COVID-19 and a control group consisting of conditionally healthy individuals. Individual studies compare transcriptomic profiles across various clinical forms: asymptomatic, mild, moderate, severe, critical, as well as across different outcomes, including convalescent patients and fatal cases. In addition, the differences between coronavirus infection and other viral and non-viral acute respiratory infections, bacterial pneumonia, asthma and COPD, sepsis, pulmonary fibrosis, and comorbidities are being investigated (Blanco-Melo et al., 2020; COvid-19 Multi-omics Blood ATlas (COMBAT) Consortium, 2022). The analysis may also include different groups of COVID-19 patients by gender, age, population, high and low viral load, depending on the time of onset of symptoms and time of sampling, groups of patients without treatment and with specific treatment, as well as different strains of SARS-CoV-2 (Fig. 1b).

In most COVID-19 transcriptomics studies, peripheral blood serves as the primary source for identifying biomarkers of the disease. Changes in blood transcriptomic profiles can be caused by effects of immunogenic factors and/or changes in immune cell proportions (Chaussabel et al., 2010). The general dysregulation of certain genes may imply a specific mechanism of immune response. To identify the most reliable biomarkers in this study, a search was conducted for overlapping differentially expressed genes (DEGs) across several independent studies of moderate and severe forms of COVID-19. This may reduce the probability that the observed expression pattern is caused by the heterogeneity of the cell population (Song et al., 2017).

Keywords and expressions such as “severe COVID-19”, “moderate COVID-19”, “mild COVID-19”, “RNA-sequencing”, “bulk RNA-sequencing” were used when searching in the PubMed system for studies that obtained data on differential gene expression in severe and moderate COVID-19 using RNA sequencing. So, the criteria for selecting studies were the analysis of the whole blood transcriptome obtained by sequencing RNA from the pool of blood cells (bulk RNAseq) in certain comparison groups – severe and moderate/mild COVID-19 (“severe versus moderate/mild”). The analysis included works that exactly meet the criteria; their characteristics are presented in Table 1.

**Table 1.**
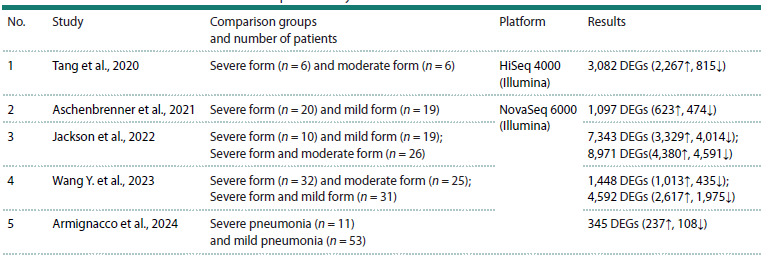
Studies that conducted a whole transcriptome analysis of severe and mild/moderate forms of COVID-19 Notes. DEGs – differentially expressed genes. ↑ – upregulation. ↓ – downregulation

**Fig. 2.**
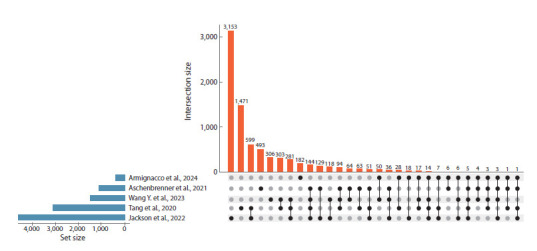
UpSet plot, showing the number of differentially expressed genes when comparing the results of the selected studies The bar graph located on the left shows the number of detected DEGs for each study. The circles that make up the matrix represent sections of the Venn diagram. The connected circles indicate the intersection of genes between certain studies. The bar graph above the matrix shows the number of unique or overlapping DEGs. For example, the first and second columns of the chart reflect the number of unique genes for research (Jackson et al., 2022) (3,153 DEGs) and (Tang et al., 2020) (1,471 DEGs). The third column shows the number of overlapping genes for this pair only (599 DEGs). For the (Jackson et al., 2022; Wang Y. et al., 2023) studies, overlapping DEGs were included, identified when comparing severe and non-severe forms according to the WHO severity classification. The diagram is constructed using https://intervene.shinyapps.io/intervene/ (Khan, Mathelier, 2017).

A total of 7,650 differentially expressed genes were identified in these studies when comparing groups of severe and moderate forms (Fig. 2). For each pair of studies, the number of overlapping genes ranges from 23 (Aschenbrenner et al., 2021 and Armignacco et al., 2024) to 1,102 (Tang et al., 2020; Jackson et al., 2022).

When analyzing the generality of the DEGs, low replication of the results was noted. Only five genes were found, which were identified when comparing severe and moderate forms of COVID-19 in each of the selected studies. These observations may indicate the significance of changes in the expression of the identified genes in the pathogenesis of severe COVID-19.

## Characterization of the most significant genes

Let us take a closer look at the characteristics of five overlapping differentially expressed genes: CD177, PPARG, PCOLCE2, SLC51A (upregulated) and ADAMTS2 (downregulated in the study (Armignacco et al., 2024) and upregulated in studies (Tang et al., 2020; Aschenbrenner et al., 2021; Jackson et al., 2022; Wang Y. et al. al., 2023).The PPARG gene encodes the nuclear receptor PPARγ, activated by the peroxisomal proliferator gamma. PPARγ regulates the peroxisomal pathway of beta-oxidation of fatty acids, as well as macrophage activation by inhibiting the production of inflammatory cytokines by monocytes (https:// www.genecards.org/).According to the DisGeNET database, this gene is significantly associated with adult-onset diabetes mellitus and obesity, for which the gene-disease association score (GDA score) is 1. These pathologies are risk factors for severe COVID- 19 (de Seabra Rodrigues Dias et al., 2022). In addition, an association with COVID-19 was found with a GDA score of 0.3 (https://disgenet.com/).

It is assumed that SARS-CoV-2 suppresses the expression of PPAR in the lungs and disrupts the anti-inflammatory mechanism of NF-κB, thereby causing a hyperinflammatory reaction in patients with severe COVID-19 (Desterke et al., 2020; Hasankhani et al., 2024). In transcriptomic studies, various changes in its expression are observed, including different tissues. For example, in the study (Vlasov et al., 2021), PPARG expression was increased in patients with an unfavorable outcome. The authors suggested that elevated PPARγ levels may be a sign of unresolved inflammation in conditions of lipid depletion, characteristic of the severe form of COVID-19 (Pei et al., 2021). According to the results of studies using network approaches, the PPARG gene has been proposed as a promising therapeutic target for controlling inflammation in COVID-19 (Auwul et al., 2021; Oh et al., 2021).

SLC51A. The alpha organic solute transporter encoded by the SLC51A gene is the main component of the OSTα/OSTβ complex, which acts as a transporter responsible for the export of bile acids from enterocytes

According to the DisGeNET database (https://disgenet. com/), SLC51A is significantly associated with primary biliary cirrhosis (GDA score = 0.65), Byler’s syndrome (GDA score = 0.5) and with a disorder associated with diabetes mellitus (GDA score = 0.4).

It has been revealed that SLC51A is a target for drugs in the treatment of COVID-19 (Morselli Gysi et al., 2021). In addition, SLC51A is included in significant genes in the differentiation of patients with sepsis and the determination of its endotypes, the clinical characteristics of which are often discussed as common with severe COVID-19 (Baghela et al., 2023; Fang, Ma, 2023). In the work (Pei et al., 2021), it was revealed that in SARS-CoV-2 infected respiratory tract and alveolar organoids derived from human embryonic stem cells, the SLC51A transporter was found in reduced amounts. Despite the importance of this gene in differentiating patients and identifying therapeutic targets for COVID-19, the role of the SLC51A gene in the mechanisms of severity of coronavirus infection remains poorly understood.

The CD177 gene mediates TNF-alpha-induced neutrophil activation, including degranulation and superoxide production, and promotes neutrophil adhesion (https://www.genecards.org/).

According to the DisGeNET database, this gene is significantly associated with myeloproliferative disorders (GDA score = 0.35), as well as with COVID-19 with a GDA score of 0.25 (https://disgenet.com/).

The relevance of this biomarker in predicting the severe course of coronavirus infection has been revealed in several transcriptomic and proteomic studies (Derakhshani et al., 2021; Lévy et al., 2021; Meizlish et al., 2021; Schimke et al., 2022; Wang Q.S. et al., 2022; Lei, 2024). In the work (Lévy et al., 2021), a high expression of CD177 and a higher average level of this serum protein in blood were observed in critically ill patients. It is assumed that neutrophil degranulation causes endothelial damage and, consequently, thrombotic complications in COVID-19 (Reusch et al., 2021). Thus, hyperexpression of CD177 is a sign of the physiopathology of COVID-19 and may act as a possible prognostic factor for the progression of the disease.

PCOLCE2 encodes a procollagen C-endopeptidase enhancer 2 protein, which provides collagen and heparin binding activity.

Increased expression of the PCOLCE2 gene is detected in patients with severe COVID-19 (Alqutami et al., 2021; Che et al., 2022). The enrichment analysis revealed that this gene is involved in the extracellular matrix organization and regulation of the inflammatory response. It is noted that PCOLCE2 can enhance the activity of collagen, and SARS-CoV-2 infection causes an increase in the level of collagen 1 in organoids and promotes the activation of fibrosis signaling pathways (Jansen et al., 2022). It is also assumed that PCOLCE2 is a factor stimulating the production of reactive oxygen species by neutrophils and the formation of neutrophil extracellular traps (NETs) (Yoon et al., 2022).

The ADAMTS2 gene encodes a member of the ADAMTS protein family, which plays a key role in the conversion of procollagen fibrillar precursors into collagen molecules (https:// www.genecards.org/).

According to the DisGeNET database, ADAMTS2 is significantly associated with the development of malignant mesothelioma (GDA score = 0.4), as well as with COVID-19 with a GDA score of 0.25 (https://disgenet.com/).

ADAMTS proteins are involved in extracellular matrix remodeling, which could potentially be important in the development of pulmonary fibrosis seen in patients with severe COVID-19. DEG analysis and analysis of gene ontology (GO) in groups of severe, precritical and critical COVID-19 showed that fibrosis-related genes (AREG, EREG, the IL-18 cytokine gene) and ADAMTS2 are highly expressed in monocytes (Zhang Y. et al., 2022).

It is assumed that ADAMTS2 mediates the pathway of TGF-β, transforming growth factor-β (de Seabra Rodrigues Dias et al., 2022). Disruption of TGF-β signaling contributes to excessive extracellular matrix deposition in tissues, which may be caused by infection and inflammation (Togami et al., 2017; Deng et al., 2024). In addition, TGF-β is involved in maintaining immune homeostasis by suppressing the activity of immunocompetent cells: this cytokine prevents the differentiation of naive T cells into classical effector T cells, suppresses the expression of cytotoxic factors and the maturation of NK cells (Deng et al., 2024). It is also assumed that TGF-β is a key cytokine regulating the chronic immune response in severe COVID-19 (Ferreira-Gomes et al., 2021).

Due to the association of ADAMTS2 with the activation of the TGF-β pathway and the formation of the extracellular matrix, increased expression of this gene in severe COVID-19 may reflect immunopathological processes characteristic of the progression of the disease and the development of pulmonary fibrosis.

**Fig. 3.**
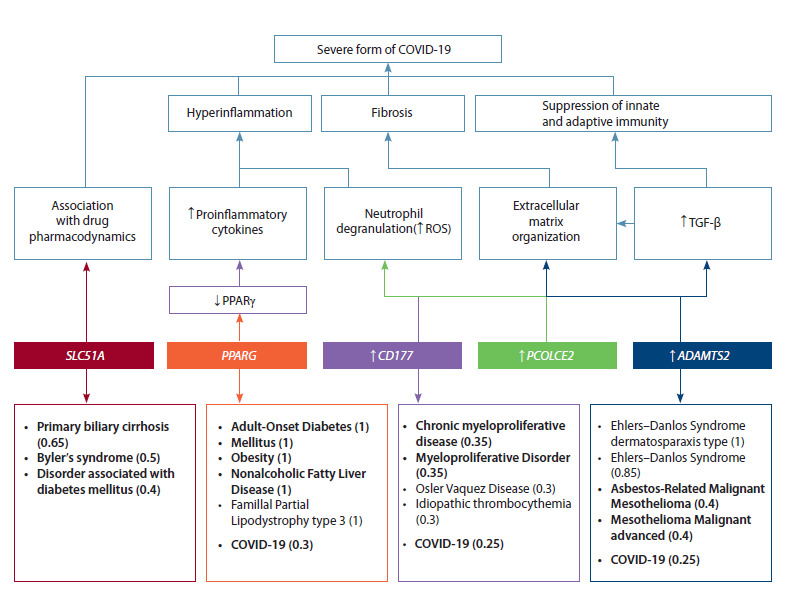
Pathogenetic aspects of severe COVID-19, including shared genes found in five selected whole transcriptome studies of moderate and severe forms of coronavirus infection and the most significant associations of these genes with diseases according to the DisGeNET database (GDA score ≥ 0.3). Diseases belonging to pathology groups that are risk factors for severe COVID-19 are highlighted in bold. ROS – reactive oxygen species.

So, the proposed mechanisms by which the identified genes are involved in the pathogenesis of severe COVID-19 were considered. They are reflected in the general scheme (Fig. 3), which also includes the most significant associations of these genes with diseases according to the DisGeNET database. A decrease in PPARG expression can lead to low PPARGγ expression, which leads to increased proinflammatory reactions; an increase in CD177 expression leads to neutrophil degranulation and the release of reactive oxygen species, and then, to the development of hyperinflammation characteristic of the severe form. Involvement of neutrophils in the pathogenesis is also possible through increased expression of PCOLCE2 as a factor that also stimulates the production of reactive oxygen species. PCOLCE2 and ADAMTS2 may be involved in the development of pulmonary fibrosis as a complication of severe pneumonia through various extracellular matrix organization pathways by activating collagen synthesis and the TGF-β pathway. Changes in ADAMTS2 expression can also lead to dysregulation of TGF-β and, thus, cause suppression of both innate and adaptive immunity. Interestingly, according to DisGeNET, these genes are associated with diseases identified as risk factors for severe COVID-19 (Prevention… COVID-19, 2023): diabetes mellitus and its associated complications (PPARG, SLC51A), obesity (PPARG), cancers (CD177, ADAMTS2) and chronic liver diseases (SLC51A).

The mechanisms driving the progression from moderate to severe COVID-19 are not yet fully elucidated, and the specific roles of these genes in the pathogenesis of severe symptoms require further investigation. Furthermore, discordant expression patterns observed across studies may indicate different regulatory signatures. Analyzing the signaling pathways involving these genes provides a novel perspective on the pathogenesis of severe SARS-CoV-2 infection, accounting for the functional relationships between genes.

## Functional enrichment analysis of overlapping differentially expressed genes associated with the severity of COVID-19

Functional enrichment was also analyzed in each of the reviewed studies for DEGs obtained by comparing severe and moderate forms of coronavirus infection. In all studies, significant signaling pathways associated with gene upregulation are associated with the inflammatory immune response of the organism. For example, the processes of degranulation and activation of neutrophils. The lymphocytic immune response pathways have been enriched with both upregulated and downregulated genes in various studies. Significant enrichments have been identified in the pathways associated with type 2 T-helper cells involved in the humoral immune response (Jackson et al., 2022), and in the processes associated with lymphocyte activation, proliferation, and regulation (Tang et al., 2020). At the same time, the processes enriched with genes with a decreased expression did not overlap between the studies. Thus, questions arise about the mechanisms of disruption of the interaction of innate and adaptive immune responses and the transition from a “viral” response in the mild form of COVID-19 to a severe inflammatory process (Jackson et al., 2022).

Since most of the significant signaling pathways in the analyzed studies are different when comparing severe and moderate forms of coronavirus infection, we performed an enrichment analysis for overlapping differentially expressed genes in order to identify common patterns of development of the severe form. Four papers were selected (Tang et al., 2020; Aschenbrenner et al., 2021; Jackson et al., 2022; Wang Y. et al., 2023), in which the identified differentially expressed genes overlapped the most. They included 149 DEGs: 102 genes with hyperexpression and 47 genes with hypoexpression. The functional enrichment analysis was performed using the WebGestalt resource (https://www.webgestalt.org/), as well as Reactome (Fabregat et al., 2017).

The results of the functional enrichment analysis using Reactome are presented in Table 2

**Table 2.**
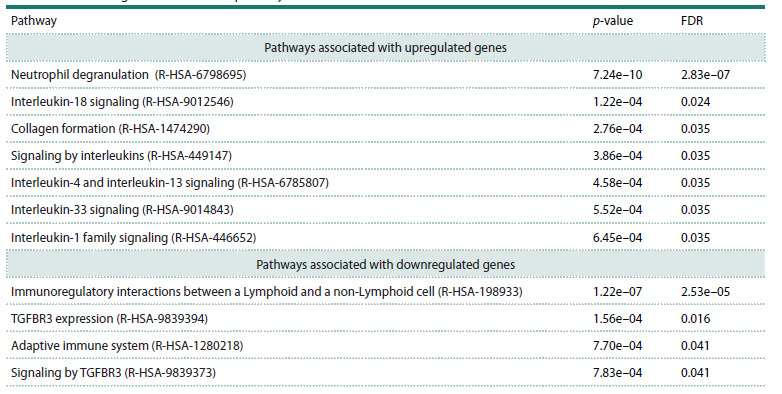
The most significant Reactome pathways with FDR ≤ 0.05

The upregulated genes in the severe disease group are associated with neutrophil degranulation (R-HSA-6798695) and collagen formation (R-HSA-1474290). Activation of interleukin signaling pathways (R-HSA-449147) – IL-18 (R-HSA-9012546), interleukin-1 family (R-HSA-446652), including IL-33 (R-HSA-9014843), IL-4 and IL-13 (RHSA- 6785807) – was also revealed. IL-4, the key cytokine of the Th2 immune response, alongside IL-13, is mainly associated with fibrotic inflammatory remodeling (Vaz de Paulaet al., 2020; Wang F. et al., 2020). In addition, IL-33, a signaling protein that warns the immune system of damage, may play an important role in all stages of COVID-19 (Zizzo, Cohen, 2020), and may also be associated with the development of fibrosis (Uchasova et al., 2018). It has been reported that IL-18 signaling, involved in the regulation of the Th1, Th2, and Th17 types of immune response, reflects inflammasome activation associated with the severity of the disease in the lungs (Filbin et al., 2021; Nasonov, Avdeeva, 2022).

Downregulated genes in severe COVID-19 were associated with adaptive immunity (R-HSA-1280218), as well as with the expression (R-HSA-9839394) and signaling of TGFBR3 (R-HSA-9839373), a type III TGF-β receptor that inhibits TGF-β signaling (Ahn et al., 2009; Chu et al., 2010). The role of this pathway was considered in the characterization of the ADAMTS2 gene.

Thus, both the analysis of pathways identified in certain studies and the analysis of pathways associated with shared genes reveal unbalancing changes in the immune response during the transition to severe form, characterized by activation of pathways of neutrophil degranulation, signals of proinflammatory (IL-1 β, IL-18) and anti-inflammatory (IL-4/13) cytokines, as well as suppression of the adaptive immune response, which may be a key factor influencing the severity of COVID-19.

**Fig. 4.**
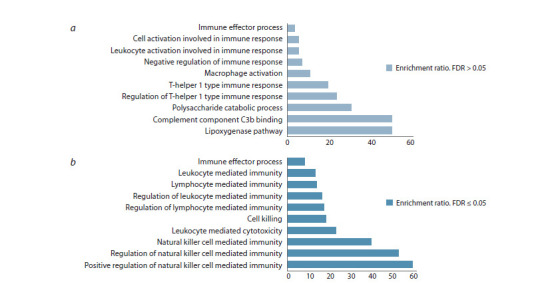
Gene Ontology pathways enriched in upregulated (a) and downregulated (b) genes

At the same time, the results of the Gene Ontology analysis (Fig. 4) included significant signaling pathways associated with downregulated genes that belong to the functional categories of NK cell mediated immunity (GO:0002228), lymphocyte mediated immunity (GO:0002449), and leukocyte mediated cytotoxicity (GO:0001909). These observations indicate the important involvement of immune cells in the response to SARS-CoV-2.

## Research designs

In order to determine the causes of variation and low replication of genes and signaling pathways, a comparison of research designs was carried out, including a comparison of the characteristics of patient cohorts, RNA sequencing platforms, as well as statistical data processing methods. Further, studies (Tang et al., 2020; Aschenbrenner et al., 2021; Jackson et al., 2022; Wang Y. et al., 2023; Armignacco et al., 2024) will correspond to the numbers 1, 2, 3, 4 and 5, respectively.

Analysis tools. Different software packages of the R environment were used to obtain differentially expressed genes: DESeq2 (Love et al., 2014), edgeR (Robinson et al., 2010) and limma (Ritchie et al., 2015). An adjusted p-value of less than 0.05 was considered significant for all studies. The threshold for absolute log2 fold change (|log2FC|) was set at ≥1 in analysis (1) and >1.5 in analyses (4, 5).

Severity of COVID-19. The inclusion of patients in the analyzed studies followed certain general criteria: age 18 years and older; positive result of the RT-PCR test for SARS-CoV-2 in respiratory samples (nasal/pharyngeal swab/sputum/ bronchoalveolar lavage) and/or serological test (5) and/or diagnosis of COVID-19 based on the presence of typical clinical symptoms and CT results (2, 5). Patients in all studies were classified as having a mild, moderate, or severe form of the disease. The classification of the severity of COVID-19 in four papers was based on the recommendations of the World Health Organization. However, the rules for the formation of severity groups were different. The mild severity corresponded to the WHO 1–2 in the study (3) and the WHO 1–4 in (2, 4). The moderate severity corresponded to the WHO level 3–4 in (3), and to the WHO level 5 in the study (4). Severe COVID-19 corresponded to WHO severity levels 5–7 in (2), WHO 5–8 in (3) and WHO 6–9 in (4). WHO Severity Classification Score is given in (3). In study (5), the severity of patients was determined by the course of pneumonia development. The patients’ condition was classified as mild, moderate, or severe pneumonia, depending on the onset and development of COVID-19 complications, including the duration of hospitalization, the need for oxygen, mechanical ventilation, or extracorporeal membrane oxygenation. Due to the variation in the formation of comparison groups, the DEG analysis included groups representing the mild form of the diseaseCollecting material. The total collection period for all works was one year, from February 2020 to February 2021. During this period, such variants of SARS-CoV-2 as Beta, Alpha, Delta, Gamma, etc. circulated (https://www.who.int/ ru/activities/tracking-SARS-CoV-2-variants). It is assumed that the virus variant may affect the severity of the disease. Transcriptomic studies were conducted to identify differences in the host response to infection with different SARS-CoV-2 variants: “Pre-VOC” and “VOCs” (variants of concern). “Pre-VOC” infection was characterized by moderate manifestations and persisted much longer than infection during VOCs circulation. Delta infection was severe, leading to high rates of hospitalization and mortality (Hughes et al., 2023; Maurya et al., 2023). It has been demonstrated that this variant is capable of enhanced replication due to sustained suppression of the innate immune response of the host organism, which potentially contributes to severe symptoms and long-term recovery (Laine et al., 2022). Despite the identification of differentiated transcriptomic responses, data on the effect on the severity of a particular variant of SARS-CoV-2 still remain contradictory. In some studies, at a significance level of 0.05, no association was found between viral lines and the severity of the disease at an early stage of the pandemic (Parikh et al., 2022).

Patients’ blood samples were collected at different time periods: during the first 24 hours after hospitalization (2, 5) or 5–7 days after admission to the hospital (3). In addition blood was collected not only in a hospital environment. For example, in study (3), blood samples were collected from patients with mild severity at home. Blood collection for further RNA isolation was carried out in PAXgene™ tubes and, after the necessary manipulations, stored at a temperature of –80 °C; the type of tube was not disclosed in the study (1). It is important to note that incomplete convergence of the results is observed when comparing the gene expression profiles of blood cells collected in different types of RNA stabilization tubes (Menke et al., 2012).

Criteria for the formation of a patient sample. The criterion for excluding patients for all studies was identification of coinfection (for example, human immunodeficiency virus). Study (2) excluded neutropenia, hematological malignancies and/or active chemotherapy, solid organ transplantation, autoimmune diseases, and any previous use of immunosuppressants (corticosteroids, anti-cytokine biologics, and biological response modifiers), while study (3) accounted for immunomodulatory treatment as a covariate in its analysis.

When comparing the formed groups, in some studies, there were no statistically significant differences in gender (1, 2, 4, 5) and age (1, 2, 4). However, study (3) reported a correlation between severity and gender/age. Additionally, the study populations varied: East Asia, Australia and Europe

Comorbidities. The analysis also considered such an important factor for the progression of the severity of COVID-19 as comorbidities. In study (2), the Charlson comorbidity index was calculated for each of the comparison groups (Charlson et al., 1987). According to this index, there were no statistically significant differences between the groups of mild, moderate and severe severity. In study (3), the incidence of endocrine comorbidities, smoking, and obesity was highest in the group with severe disease: 70, 50, and 50 %, respectively, compared with 53.8, 15.4, and 46.2 % of patients with moderate COVID and 21, 5.3, and 5.3 % with mild COVID-19. In study (5), significant differences between patients of varying severity among comorbidities were identified only for diabetes mellitus (adjusted p = 0.048).

Thus, despite meeting the criteria for selecting studies, noticeable differences were found in the characteristics of the comparison groups, as well as the analysis tools, which may explain the observed variation in the results of the selected studies. Another key factor influencing the variability of the results is the cellular heterogeneity of the analyzed samples. It is possible to study the contribution of cellular heterogeneity and consider in more detail the composition and functional characteristics of cells using the single-cell RNA sequencing approach (scRNA-seq).

## Single-cell RNA sequencing in studying severe COVID-19

Numerous studies have recently employed single-cell RNAseq technology to characterize the immune landscape in COVID- 19 patients. Table S1 (Supplementary materials)1 shows the features of gene expression and cellular composition when comparing severe and moderate forms of COVID-19. According to the results of scRNA-seq of blood cells, the severe form is characterized by certain changes in the composition of immune cells: there was a decrease in plasmocytoid dendritic cells, NK cells, non-classical monocytes and an increase in the proportion of classical monocytes, mature neutrophils and immature subpopulations of monocytes and neutrophils. In the severe form of COVID-19, low-density neutrophils (LDN) associated with dysfunctional immune responses have also been found to occur in conditions of emergency myelopoiesis (Schultze et al., 2019), which was not observed in the mild form. LDN, like mature neutrophils, secreted high levels of alarmins S100A8 and S100A9 (Schulte-Schrepping et al., 2020; Silvin et al., 2020; Ren et al., 2021; Wilk et al., 2021). Alarmins are released under inflammatory conditions and form a stable heterodimer known as “calprotectin” (Wang S. et al., 2018), which is involved in the activation and chemotaxis of neutrophils (Ryckman et al., 2003), and is also suspected to be the cause of cytokine release syndrome (Silvin et al., 2020). However, in severe cases, multidirectional gene expression of certain proinflammatory (IL1B, TNF) and anti-inflammatory (IFNG) cytokines was also detected, with an overall increased production of proinflammatory cytokines by immune cells

Supplementary Materials are available in the online version of the paper: https://vavilov.elpub.ru/jour/manager/files/Suppl_Gusar_Engl_30_1.pdf


The revealed differences in the composition and functional activity of T cells with varying severity and at different stages of COVID-19 may indicate the complexity of T cell responses to infection (Ren et al., 2021). At the same time, each type of cell involved in the response to coronavirus infection differs in a specific pattern of gene and membrane protein expression, reflecting the complex pathophysiology of severe COVID-19 manifestations. The main differences were found in the response of cells to type I interferon, as well as in the expression of interferon-stimulated genes (ISG15 and IFITM1/2). Such observations can be explained by temporary changes at different stages of disease progression. In severe cases, there is a time shift: from an early but short-term reaction to type I interferon to a proinflammatory reaction at later stages (Arunachalam et al., 2020); therefore, it is crucial to account for the timing of sample collection relative to symptom onsetduring the analysis. However, not all studies specify cell types when analyzing gene expression, which also makes it more difficult to identify common changes in severe COVID-19.

Several studies have also observed shared pathophysiological pathways driving severe disease, reflecting the phenomenon of dysregulation of the immune response. For example, most studies have revealed a disrupted major histocompatibility complex class II antigen presentation by monocytes to T cells (HLA-DR), which indicates the development of immunosuppression (Zurochka et al., 2008). In addition, an activated pathway of NF-κB, a transcription factor contributing to the development of a “cytokine storm” and oxidative stress by enhancing the synthesis of proinflammatory cytokines and reactive oxygen species (ROS) by activated macrophages and neutrophils, was identified (Bolevich S.B., Bolevich S.S., 2020; Kesika et al., 2024). Oxidative stress resulting from increased ROS formation and decreased antioxidant protection contributes to the pathogenesis of the severe form of COVID-19, leading to damage to cells and tissues through direct damage, lipid peroxidation and protein oxidation. Oxidative stress, coupled with cytokine release, induces endothelial dysfunction and activates the coagulation cascade, leading to microvascular thrombosis. Proinflammatory cytokines additionally stimulate ROS synthesis, exacerbating ARDS and lung tissue damage, causing a vicious cycle between oxidative stress and a cytokine storm (Polonikov, 2020; Gadotti et al., 2021; Alam, Czajkowsky, 2022; Labarrere, Kassab, 2022). The analysis of transcriptomic data also confirms the importance of a disturbance of the redox balance in the pathogenesis of severe COVID-19. The authors of the study (Saheb Sharif-Askari et al., 2021) conducted an in silico analysis of publicly available transcriptomic data from COVID-19 patients to assess the expression levels of 125 genes associated with oxidative stress. Seven genes (MPO, S100A8, S100A9, SRXN1, GCLM, SESN2 and TXN) were found to have increased expression in whole blood and lung autopsies of patients with severe COVID-19 compared with patients with a non-severe form. In the work (Tavassolifar et al., 2023), increased levels of intracellular ROS, decreased levels of glutathione, and upregulation of oxidant and antioxidant genes were detected in peripheral blood mononuclear cells of patients with COVID-19 (CAT, NFE2L2, SOD1, SOD2 and CYBB).

## Commonality of bulk and single-cell RNA-seq studies

Some patterns revealed by analyzing the transcriptomes of bulk blood samples of patients with moderate and severe forms of COVID-19 were also replicated using single-cell transcriptomics

An increase in the proportion of circulating neutrophils, their hyperactivation and dysregulation, and the appearance of immature or developing neutrophils associated with the severity of COVID-19 have been found in several studies using the scRNA-seq approach. The analysis revealed hyperexpression of genes encoding proinflammatory cytokines associated with phagocytosis and degranulation, as well as genes (for example, PADI4, MPO, ELANE, and PRTN3) that are involved in the formation of neutrophil extracellular traps (Barnes et al., 2020; Schulte-Schrepping et al., 2020; Silvin et al., 2020; Wilk et al., 2021). Excessive NET release from neutrophils contributes to the development of oxidative stress, hypercoagulation, impaired alveolar microcirculation and damage to lung tissue. The levels of components of neutrophil extracellular traps – for example, DEFA1 RNA and neutrophil elastase (ELANE) activity in the blood – are considered as potential biomar- kers of the severe form of COVID-19 (Wargodsky et al., 2022).

In addition, activated mature neutrophils in severe COVID- 19 showed increased expression of the CD177 gene, the possible role of which in pathogenesis was previously discussed, as well as hyperexpression of the CD274 gene and the ARG1 gene (Schulte-Schrepping et al., 2020; Wilk et al., 2021), which is part of 149 overlapping DEGs. CD274 (PD-L1) and ARG1 are associated with suppression of T cell activation, suggesting that neutrophils may perform immunosuppressive functions in severe COVID-19 (Schulte-Schrepping et al., 2020). These observations are consistent with suppression of shared lymphocyte signaling pathways.

The enrichment of both pro- and anti-inflammatory interleukin signaling pathways identified by bulk RNA-seq analysis is consistent with the detected high cytokine expression in various immune cells, mainly classical monocytes and macrophages via single-cell analysis approach. Increased levels of proinflammatory cytokines are thought to play an important role in the severe progression of COVID-19, causing a hyperinflammatory reaction called a “cytokine storm” (Ershov et al., 2020; Guo et al., 2020). It was found that the IFN-I reaction can contribute to the development of the hyperinflammatory response caused by IL-1β in severe progression of COVID-19 (Lee J.S. et al., 2020). The genetic signature of the IL-4/13 and IL-18 signaling pathways in monocytes also increased significantly in severe cases (Lee J.S. et al., 2020; Liu C. et al., 2021; Jeong et al., 2023).

Natural killer (NK) cells play an important role in innate immune responses to viral infections. Decreased functional activity of NK cells is associated with acute and chronic viral infection (Abakushina et al., 2012). Analysis of single-cell RNA sequencing results revealed differences in the transcriptome of NK cells between groups of patients with moderate and severe COVID-19. For example, a significant transcriptional remodeling was driven by an increase in the expression of canonical NK cell activation genes in the severe form, including increased expression of genes encoding cytotoxic effector molecules GZMB, PRF1, GZMA, as well as the proliferation marker MKI67 (Wilk et al., 2021; Shaymardanov et al., 2022). However, genes associated with NK cell cytotoxicity (GZMM, GZMH, GZMA) were identified among the common low-expression genes in bulk RNA-seq analysis, and the NK cell-mediated immunity signaling pathway (GO:0002228) was enriched in downregulated genes. At the same time, signs of NK cell depletion at the transcriptome level in patients with the severe form were observed in scRNA-seq studies (Lee J.S. et al., 2020; Krämer et al., 2021; Liu C. et al., 2021; Wilk et al., 2021; Witkowski et al., 2021). In the work (Witkowski et al., 2021) NK cells demonstrated dysregulation of cytokine production, cell-mediated cytotoxicity, and response to the virus, despite high expression of cytotoxic effector molecules. At the same time, transcriptional networks responsible for the activation of NK cells were superimposed by the dominant signature of the response to TGF-β. In the study (McClain et al., 2023) differential expression of the TGFB1 gene encoding transforming growth factor beta-1 was observed in CD14+ monocytes and was associated with a worsening of COVID-19, and in the study (Ren et al., 2021) upregulated expression of TGFB1 was observed in T cells, B cells, NK and dendritic cells. The TGF-β pathway was identified as one of the key pathways activated in severe cases of SARS-CoV-2 Delta infection (Shaymardanov et al., 2022). Thus, the involvement of TGF-β is confirmed by the analysis of the transcriptome at the level of a single cell. These results suggest that disturbances in the cytotoxicity of NK cells, including through the influence of the TGF-β pathway, may be associated with the mechanisms of severe COVID-19 development (Su et al., 2020; Lee M.L., Blish, 2023).

**Fig. 5.**
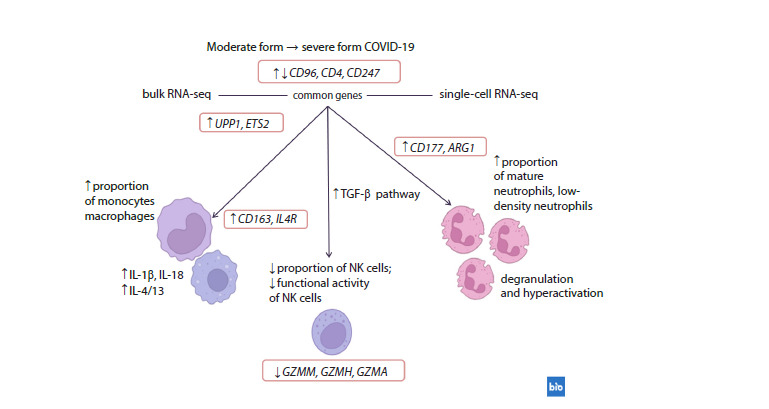
Shared differentially expressed genes and consistent signatures identified through the comparison of moderate and severe COVID-19 cases using bulk and single-cell RNA-sequencing approaches. ↑ – increase in proportion or expression; ↓ – decrease in proportion or expression; ↑↓ – multidirectional expression. Made with https://BioRender.com.

The features of the development of the severe form of COVID-19 are characterized by a multifaceted immune dysregulation, which is described as a state of imbalance (Yao et al., 2021). Despite the differences in RNA-sequencing methodologies, shared differentially expressed genes and consistent patterns were identified when comparing moderate and severe forms (Fig. 5). There is a dysregulation of innate (hyperinflammatory reactions, decreased cytotoxicity of NK cells, activation and degranulation of neutrophils) and adaptive (suppression of lymphocytic immunity pathways) immune responses. At the same time, the multidirectional expression of some shared DEGs, for example CD96, CD4 and CD247, expressed on different immune cells and involved in the immune response, was also revealed, which is consistent with the phenomenon of cellular heterogeneity. In bulk RNA-seq, the overall expression levels are influenced by shifts in cell type proportions within the patients’ blood samples, while in single-cell RNA-seq, gene expression levels are obtained at single-cell resolution.

## Conclusion

Peripheral blood transcriptomics is a powerful tool for studying the immune system in patients with coronavirus infection. The dynamics of the organism’s immune response during an infectious process is reflected in changes in gene expression and can be studied in patients with severe disease development to identify key pathways for disease progression. It is assumed that changes in the expression of certain genes may be predictors of severe COVID-19.

So, this work analyzes the core set of differentially expressed genes and signaling pathways identified across COVID-19 transcriptomic studies. Studies that met the selection criteria were analyzed, including bulk RNA-seq of whole blood and a comparison of moderate and severe cases. A total of 7,605 differentially expressed genes were identified; five overlapping genes were detected: CD177, PPARG, PCOLCE2, SLC51A and ADAMTS2. Their possible role in the pathogenesis of severe COVID-19 was considered. Pathological processes such as hyperinflammation, fibrosis, and dysregulation of adaptive and innate immunity may be among the possible mechanisms of progression of coronavirus infection. For the four papers closest in design, shared enriched pathways for 149 DEGs were found, which included activation of neutrophil degranulation, interleukin signaling pathways, collagen biosyn- thesis, and suppression of adaptive and NK cell immune pathways

The analysis also included transcriptomic studies of various clinical forms of COVID-19 using single-cell technology, which confirm the hypothesis of immune dysregulation identified by the results of bulk RNA-seq in patients with development of the severe form. The severe course is characterized by increased proportions and activity of mature and developing neutrophils, the onset of cytokine release syndrome and oxidative stress, as well as impaired cytotoxicity of NK cells involving the TGF-β pathway

Research in the field of COVID-19 transcriptomics reveals the features of cellular and molecular processes that can lead to the development of a severe form of coronavirus infection. Identification of RNA biomarkers in patients with COVID-19 can contribute to more effective recognition of patients from risk groups, stratification of disease severity, and prediction of severe course and outcomes of COVID-19. Transcriptomic biomarkers also show their effectiveness in differentiating patients with SARS-CoV-2 infection from patients with other lung infections. In addition, they play an important role in determining the directions of effective treatment of infection, including long-term COVID-19. RNA markers can be used as therapeutic targets, as well as in evaluating treatment response. These advantages highlight the potential of RNA biomarkers for clinical applications in diagnosis, prognosis, and the development of personalized treatment and rehabilitation strategies for patients.

However, despite the high sensitivity of RNA biomarkers, their integration into clinical practice faces significant difficulties. Methodological diversity of studies often makes it difficult to generalize the results and limits the possibility of identifying reproducible RNA biomarkers of COVID-19 severity. The variability of RNA sequencing data may also stem from individual physiological differences in patients and environmental factors. The results may not be representative for patients from different populations because of the population-specific immune response (Nédélec et al., 2016; Randolph et al., 2024). It is also important to consider the specifics of obtaining the material in a clinical setting: accurate measurement of RNA is hampered by the instability of RNA in the blood and the difficulty of purification. In the future, integrating new technologies such as single-cell RNA sequencing, validation of the effectiveness of the identified predictors in larger multicenter trials, and the development of standardized research protocols will help overcome some of the limitations of the clinical use of RNA markers (Schultze, Aschenbrenner, 2021; Chen et al., 2022; Wargodsky et al., 2022; Eldien et al., 2025; Shimansky et al., 2025). Furthermore, to fully elucidate the complex pathogenesis of severe COVID-19, it is also important to apply an integrated “omics” approach, which, in addition to transcriptomics, will include other methods for studying the immune response to SARS-CoV-2: proteomics, metabolomics, epigenetics, multiplex measurements of cytokines/ chemokines, etc. Based on multi-omic technologies, new approaches can be developed for outcome prediction, prevention and treatment of severe COVID-19.

## Conflict of interest

The authors declare no conflict of interest.
